# Prolactin-induced tyrosyl phosphorylation of PAK1 facilitates epithelial-mesenchymal transition

**DOI:** 10.17912/micropub.biology.001136

**Published:** 2024-04-09

**Authors:** Alan Hammer, Maria Diakonova

**Affiliations:** 1 Department of Biological Sciences, University of Toledo, Toledo, Ohio, United States

## Abstract

PAK1 and prolactin (PRL) regulate breast cancer. Prolactin-activated JAK2 tyrosyl phosphorylates PAK1 (pTyr-PAK1). We demonstrate here that pTyr-PAK1 regulates epithelial-mesenchymal transition (EMT) in breast cancer cells. PRL treatment of T47D PAK1 WT cells leads to downregulation of E-cadherin surface expression and “ectodomain shedding” (extracellular cleavage of E-cadherin). pTyr-PAK1 increases mRNA levels of Snail, Slug, and Twist2, transcriptional factors implicated in E-cadherin repression. pTyr-PAK1 also significantly increases PRL-dependent Slug activity leading to expression of vimentin, a hallmark of EMT. Thus, our current data on pTyr-PAK1 regulation of EMT bring insight into the role of PAK1 and PRL in human breast cancer.

**Figure 1. Prolactin-activated and tyrosyl-phosphorylated PAK1 increases epithelial-mesenchymal transition in breast cancer cells:**
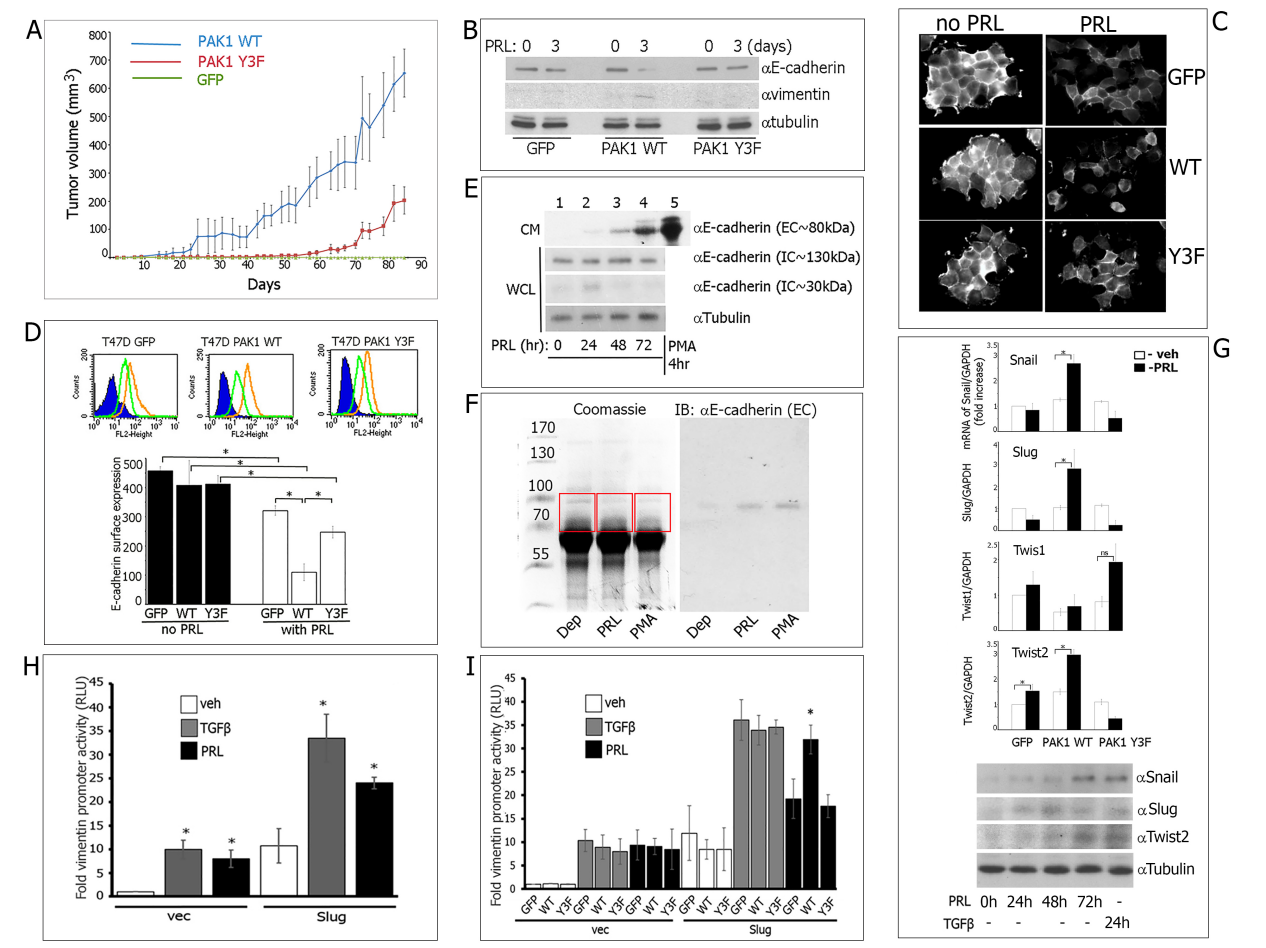
**A)** PRL-activated pTyr-PAK1 enhances tumor growth in a xenograft mouse model. Human breast cancer TMX2-28 cells stably overexpressing GFP, PAK1 WT or PAK1 Y3F were inoculated into the mammary pads of NSG mice. hPRL was injected for 8 weeks and tumor volumes were measured. **B)** PRL downregulates E-cadherin expression and promotes expression of pro-EMT marker vimentin in a pTyr-PAK1-dependent manner. Indicated clones of T47D cells were treated with PRL for 0 or 3 days. PAK1 WT, but not GFP or PAK1 Y3F clones, demonstrates decreased E-cadherin and acquired vimentin expression in response to PRL. **C)** PRL reduces surface E-cadherin in pTyr-PAK1-dependent manner. Immunofluorescence of surface E-cadherin in T47D clones treated with PRL for 0 or 3 days, fixed and labeled with anti-E-cadherin AB without permeabilization. **D)** pTyr-PAK1 WT regulates E-cadherin cell surface expression. E-cadherin cell surface expression in T47D GFP, PAK1 WT and Y3F clones treated without (yellow lines and black bars) or with (green lines and white bars) PRL was assessed by flow cytometry after staining with an extracellular anti-E-cadherin AB. Bars represent mean ± SE. *P<0.05 as compared to the cells treated with vehicle. **E-F)** PRL promotes E-cadherin cleavage. ( **E** ) T47D PAK1 WT cells were treated with PRL for 0-72 hrs. Conditioned media (CM) and whole cell lysates (WCL) were immunoblotted for the indicated AB. αE-cadherin (EC) AB is specific for the extracellular domain of E-cadherin, while αE-cadherin (IC) AB is specific for the intracellular domain of E-cadherin. ( **F** ) T47D PAK1 cells were treated as in E, and the CM was subjected to SDS-PAGE. The gel was either stained with Coomassie blue (left) or subjected to Western blotting with antibody to extracellular E-cadherin (EC; right). The bands between 70kDa and 100kDa were excised from the gel (Coomassie staining, red boxes) and analyzed using mass spectrometry. **G)** pTyr-PAK1 increases transcription and expression of Snail, Slug and Twist2 in T47D clones. The mRNA levels for Snail, Slug, Twist1 and Twist2 were analyzed by qRT-PCR. Bars represent mean ± SE. P<0.05 as compared to vehicle treatment. To assess proteins translation, PAK1 WT cells were treated with either PRL (500 ng/ml), TGFβ (5 ng/ml) or vehicle for 0, 24, 48 or 72 hours. Whole cell lysates were immunoblotted for the indicated AB. **H and I)** PRL activates Slug in a pTyr-PAK1-dependent manner. Fold vimentin promoter activity in T47D parental cells ( **H** ) or in T47D clones ( **I** ), co-transfected with either vector (vec) or Slug and treated with either vehicle (veh), PRL or TGFβ for 24h. Bars represent mean ± SE. *P<0.05 as compared to vehicle treatment ( **H** ) or to GFP cells with the same treatment ( **I** ).

## Description

Breast cancer affects one in eight women during their lives. Over 70% of cases occur in women who have no identifiable risk factors. Survival rates for breast cancer patients are significantly reduced upon tumor invasion and metastasis. In order for mammary tumor cells to invade and migrate, they must first lose their epithelial characteristics and gain the characteristics of mesenchymal cells. This transition from polar, tightly-adhered epithelial cells to non-polar, migratory and invasive mesenchymal cells is called epithelial-to-mesenchymal transition (EMT). One of the most important changes during EMT is the downregulation of cell-cell adhesion proteins, such as E-cadherin, and the emergence of mesenchymal proteins, including intermediate filament vimentin (reviewed in (Brabletz, Schuhwerk, Brabletz, & Stemmler, 2021; Pastushenko & Blanpain, 2019) ).

There is mounting evidence that peptide hormone/cytokine prolactin (PRL) plays a significant role in breast cancer. PRL is not only secreted from the anterior pituitary (reviewed in (Marano & Ben-Jonathan, 2014) ), but it is also locally produced by several target organs, including the mammary gland, prostate, skin, brain, some immune cells, adipocytes and others (extra-pituitary PRL, reviewed in (Ben-Jonathan, Liby, McFarland, & Zinger, 2002; Ben-Jonathan, Mershon, Allen, & Steinmetz, 1996) ). PRL is involved in tumor progression by increasing cell proliferation and angiogenesis, and by reducing apoptosis (reviewed in (Clevenger & Rui, 2022; Jacobson, Hugo, Borcherding, & Ben-Jonathan, 2011; Mujagic, Srabovic, & Mujagic, 2009; Schuler & O'Leary, 2022; Tworoger & Hankinson, 2006, 2008; Wagner & Rui, 2008)). High-normal circulating PRL levels increase breast cancer risk in both pre- and post-menopausal women (Tworoger, Eliassen, Rosner, Sluss, & Hankinson, 2004; Tworoger & Hankinson, 2006; M. Wang, Wu, Chai, Zhang, & Jiang, 2016) . PRL regulates breast tumor metastasis: PRL expression has been associated with cell invasion, metastasis and reduced survival (reviewed in (Carrasco-Ceballos et al., 2023; Hammer & Diakonova, 2015)). PRL administration in animal models increases metastasis, while loss of the PRL receptor (PRLR) prevents the progression of neoplasia into invasive carcinoma (Hammer & Diakonova, 2016; Liby, Neltner, Mohamet, Menchen, & Ben-Jonathan, 2003; Oakes et al., 2007) . Epidemiologic studies also linked elevated level of circulating PRL to breast cancer metastases (Bhatavdekar et al., 1990; Holtkamp, Nagel, Wander, Rauschecker, & von Heyden, 1984; Mujagic & Mujagic, 2004) .

Upon PRL binding to its receptor, PRLR activates non-receptor tyrosine kinase JAK2 and subsequent several downstream signal transduction cascades. We have implicated the serine-threonine kinase PAK1 as a substrate of prolactin-activated JAK2 (Rider, Shatrova, Feener, Webb, & Diakonova, 2007) . PAK1 is also implicated in regulation of breast cancer. PAK1 is amplified in several human cancer types, including 30-33% of breast tumor samples and cancer cell lines ( (Shrestha et al., 2012) , reviewed in (Eswaran, Li, Shah, & Kumar, 2012; Kumar, Gururaj, & Barnes, 2006; Molli, Li, Murray, Rayala, & Kumar, 2009) ). PAK1 genomic amplification at 11q13 is prevalent in luminal breast cancer and PAK1 protein expression is associated with lymph node metastasis (Ong et al., 2011) . Expression and activity of PAK1 in human breast tumors correlates with tumor grade (Holm et al., 2006; Salh, Marotta, Wagey, Sayed, & Pelech, 2002; Vadlamudi et al., 2000) , and transgenic expression of active PAK1 in mouse mammary tissue is tumorigenic (R. A. Wang, Zhang, Balasenthil, Medina, & Kumar, 2006). PAK1 plays an important role in cell proliferation, survival, cell motility and EMT (reviewed in (Kanumuri et al., 2020; Kumar et al., 2006; Kumar, Paul, Amjesh, George, & Pillai, 2020; Molli et al., 2009; Rajendran et al., 2022) ).

However, the precise role of PRL-activated PAK1 in breast cancer and the respective signaling pathways affected is not well defined. We have previously discovered that JAK2 phosphorylates PAK1 on tyrosines 153, 201, and 285 (Rider et al., 2007) . We have shown that tyrosyl phosphorylated PAK1 (pTyr-PAK1) facilitates PRL-dependent breast cancer cell motility via several mechanisms: formation of paxillin/GIT1/βPIX/pTyr-PAK1 complexes resulting in increased adhesion turnover and phosphorylation of actin-binding protein filamin A (Hammer, Oladimeji, De Las Casas, & Diakonova, 2015; Hammer et al., 2013; Rider & Diakonova, 2011) . Additionally, we have demonstrated that pTyr-PAK1, in response to PRL, regulates PTP-PEST-dependent FAK dephosphorylation, resulting in augmented breast cancer cell migration and invasion. Furthermore, we provided *in vivo* evidence that pTyr-PAK1 stimulates PRL-induced tumor metastasis in mice (Hammer & Diakonova, 2016) . pTyr- PAK1 also stimulated invasion of breast cancer cells in response to PRL and three-dimensional collagen IV (Rider, Oladimeji, & Diakonova, 2013) . We have also shown that pTyr-PAK1 increases cyclin D1 promoter activity in response to PRL (Tao, Oladimeji, Rider, & Diakonova, 2011) and synergistically activates estrogen receptor with estrogen (Oladimeji, Skerl, Rush and Diakonova, 2016) . These published data suggest that tyrosyl phosphorylation of PAK1 enhances PAK1 kinase activity and its ability to form protein/protein interactions. Both PAK1 activities are important for PRL-dependent breast cancer cell adhesion, motility, and invasion (reviewed in (Carrasco-Ceballos et al., 2023; Hammer & Diakonova, 2015)).

Here we first show that PRL-activated pTyr-PAK1 enhances breast cancer growth *in vivo* ( **Fig. 1A** ). We inoculated human breast cancer TMX-28 cells (a variant of the MCF-7 breast cancer cells (Fasco, Amin, Pentecost, Yang, & Gierthy, 2003) , stably overexpressing GFP, PAK1 WT or PAK1 Y3F (the clones were characterized previously in (Rider et al., 2013) ) into the mammary pads of NSG mice. PAK1 Y3F mutants are a phospho-tyrosine-deficient PAK1 in which Tyr(s) 153, 201, and 285 were mutated to phenylalanines (Rider et al., 2007) . The hPRL was injected every other day for 8 weeks and tumor volumes were measured. Fig. 1A demonstrates that PRL enhances tumor growth in mice injected with PAK 1 WT but not in mice injected with GFP cells. Tumor growth in mice injected with PAK1 Y3F, which is still kinase active, was strongly inhibited as compared to mice injected with PAK1 WT ( **Fig. 1A** ).

Previously, we have shown that pTyr-PAK1 plays a significant role in PRL-induced breast cancer cell motility and metastasis in mice *in vivo* as only cells overexpressing PAK1, but not PAK1 Y3F, were able to migrate from the primary tumor to the lungs (Hammer & Diakonova, 2016) . As epithelial-mesenchymal transition is the initial step for cancer invasion and metastasis, we decided to test whether PRL-activated PAK1 participates in EMT. The hallmark of EMT is the loss of E-cadherin expression (a cell-cell adhesion protein) which allows cancer cells to gain mobility, leave the primary tumor site, and invade adjacent tissues. We have shown here that PRL treatment of epithelial-like T47D cells led to significant reductions in E-cadherin in pTyr-PAK1-dependent way ( **Fig. 1B** ). Only PAK1 WT clone, in which PAK1 WT is tyrosyl-phosphorylated by JAK2 in response to PRL, was able to downregulate E-cadherin expression in contrast to PAK1 Y3F clone. In addition to downregulation of E-cadherin, PRL-activated PAK1 also contributes to upregulation of mesenchymal marker vimentin, the mesenchymal intermediate filament protein and the hallmark of EMT, while PAK1 Y3F and GFP clones failed to do so ( **Fig. 1B** ).

Next, we focused on the surface expression of E-cadherin, which is reduced during EMT (for review (Kalluri & Weinberg, 2009) ). T47D clones were treated with or without PRL for 3 days, fixed and stained for the extracellular domain of E-cadherin without the cells permeabilization ( **Fig. 1C** ). While PRL treatment decreased E-cadherin surface expression in all clones, PAK1 WT cells demonstrated a more robust reduction in E-cadherin surface expression and mesenchymal-like morphological change when compared to GFP and Y3F clones ( **Fig. 1C** ). To provide quantitative data on E-cadherin surface expression, we performed the FACS analysis ( **Fig. 1D )** . T47D clones were treated with PRL for 0 to 3 days and surface E-cadherin expression was assessed by FACS analysis using an antibody that recognizes only the extracellular domain of E-cadherin. In agreement with our data from the immunofluorescence experiments, PRL treatment significantly reduced surface E-cadherin expression in all cells; however, cells expressing PAK1 WT had significantly less surface E-cadherin when compared to both the GFP and the PAK1 Y3F expressing cells ( **Figure 1D** ). This suggests that PRL downregulates E-cadherin surface expression and that maximal downregulation requires tyrosyl phosphorylation of PAK1.

The cell surface expression of E-cadherin is regulated by either exocytosis, endocytosis, or extracellular cleavage (“ecdodomain shedding” (Reiss, Ludwig, & Saftig, 2006) ). E-cadherin ectodomain shedding was first demonstrated by the detection of a soluble 80 kDa fragment in the medium of MCF-7 cells (Wheelock, Buck, Bechtol, & Damsky, 1987) . We decided to assess the E-cadherin ectodomain shedding in T47D PAK1 WT cells treated with PRL by testing the conditioned medium (CM) for the soluble 80 kDa fragment of E-cadherin. Both the CM and the cell lysates (WCL) were subjected to SDS-PAGE ( **Fig. 1E** ). The soluble E-cadherin was detected by an antibody specific for the extracellular domain of E-cadherin (extracellular E-cadherin, EC), while the intracellular E-cadherin (IC) was detected using an antibody specific for the intracellular domain of E-cadherin. Treatment of cells with phorbol-12-myristate-13-acetate (PMA) for 4 hr was used as a positive control because this treatment causes release of the soluble 80 kDa fragment of E-cadherin (Maretzky et al., 2005) ( **Fig. 1E** , lane 5). We have detected this cleaved fragment of E-cadherin (EC~ 80kDa) in the CM in 48-72 hr of PRL treatment ( **Fig. 1E** , lanes 3 and 4). A 30kDa band recognized by anti-E-cadherin antibody specific to intracellular domain of E-cadherin (IC ~30 kDa) was detected in cell lysates after 24 hr PRL treatment, but disappeared by 48 hr, suggesting that this fragment was degraded over time ( **Fig 1E** , lanes 2 and 3).

To confirm that 80 kDa protein in the media is the cleaved E-cadherin, we treated the cells as above, and the CM was subjected to SDS-PAGE. The gel was either stained with Coomassie blue ( **Fig. 1F** , left) or subjected to Western blotting with antibody to extracellular E-cadherin (EC; **Fig. 1F** , right). The bands between 70kDa and 100kDa were excised from the gel ( **Fig. 1F** , Coomassie staining, red boxes) and analyzed by mass spectrometry (tandem mass spectrometry, MS/MS). Mass spectrometry analysis confirmed with high confidence that the bands we observed in our Western blot were E-cadherin fragments. These data suggest that PRL causes E-cadherin shedding in T47D PAK WT cells.

Several proteases have been implicated in the extracellular cleavage of E-cadherin, including MMP-3 (Noe et al., 2001). Furthermore, the reverse correlation between expression of MMP-1 and MMP-3 and expression of E-cadherin has been demonstrated in tumor cells (Nawrocki-Raby et al., 2003) . It is particularly interesting because we have previously shown that PRL-activated pTyr-PAK1 induces secretion of MMP-1 and MMP-3 in the breast cancer cells grown in 3D collagen IV (Rider et al., 2013) . It is known that MMP-1 degrades type I collagen, which is a major component of the ECM, and MMP-3 degrades collagen IV, which is a main component of basement membrane. Additionally, pTyr-PAK1-dependent secretion of MMP-3 may participate in the extracellular cleavage of E-cadherin leading to the EMT and increased invasion of breast cancer cells.

Several transcriptional factors have been implicated in the transcriptional repression of E-cadherin, including Snail, Slug, Zeb1, Zeb2/Sip1, Twist and E47 (Batlle et al., 2000; Wheelock, Shintani, Maeda, Fukumoto, & Johnson, 2008) . We have shown here that pTyr-PAK1, in response to PRL, increases mRNA levels of Snail, Slug, and Twist2 but not Twist1 ( **Fig. 1G )** . We also confirm that the protein levels of Snail, Slug and Twist2 were increased in 24 – 73 hours of PRL treatment in the cells overexpressing PAK1 WT ( **Fig. 1G** ). These data suggest that PRL promotes E-cadherin repression in a pTyr-PAK1-dependent manner via increased transcription of pro-EMT transcription factors Snail, Slug and Twist 2. One possible mechanism of PAK1-dependent regulation of these transcriptions may be a NFkB because PAK1 activates the NFkB pathway (Frost et al., 2000) , and NFkB induces Snail, Slug and Twist 2 expression (Wu et al., 2009; C. Zhang, Carl, Trudeau, Simmet, & Klymkowsky, 2006; K. Zhang et al., 2011) . Additionally, there is evidence that Snail mRNA expression is regulated by JAK2/STAT3 (Huang et al., 2011; Yadav, Kumar, Datta, Teknos, & Kumar, 2011), a major PRL-induced signaling pathway, suggesting there may be PAK1-independent role for PRL in the expression of Snail. However, we did not observe any PRL-induced Snail expression in GFP cells ( **Fig. 1G** , left bars).

It has been shown that PAK1 phosphorylates Snail at Ser246, resulting in Snail accumulation in the nucleus. Snail phosphorylation by PAK1 retained Snail in the nucleus and promoted Snail-mediated repression of E-cadherin and occluding (Yang et al., 2005) . We have previously shown that nuclear translocation of PAK1 is ligand-dependent: PRL treatment activates and induces translocation of PAK1 into nucleus while estrogen activates PAK1 only in the cytoplasm. Moreover, tyrosyl phosphorylation of PAK1 is essential for this nuclear translocation because Y3F mutant is retained in the cytoplasm in response to PRL (Oladimeji and Diakonova, 2016) . Since Slug is present exclusively in the nucleus (Dominguez et al., 2003) , it is important to notice that PRL stimulates PAK1 kinase activity in the nuclear fraction (Oladimeji and Diakonova, 2016) . Thus, we can hypothesize here that, in response to PRL, PAK1 WT can phosphorylate Snail at Ser246 (our ongoing project) in cytoplasm and nucleus and promote its nuclear translocation to a higher extent than PAK1 Y3F, which is still kinase active but retains in the cytoplasm. In support, tyrosyl phosphorylation of PAK1 by JAK2 increased PAK1-mediated Snail phosphorylation and induced EMT in response to irradiation of lung cancer cells (Kim et al., 2014) .

Slug, a transcription factor in the same family as Snail, also binds to the E-cadherin promoter and represses E-cadherin transcription (reviewed in (Phillips & Kuperwasser, 2014) ). Slug also upregulates transcription of vimentin, the mesenchymal intermediate filament protein and a hallmark of EMT (Vuoriluoto et al., 2011) . Because pTyr-PAK1 stimulates expression of vimentin ( **Fig. 1B** ), we asked whether pTyr-PAK1, in addition to increased transcription of Slug ( **Fig. 1G** ), is also involved in Slug activation and regulation of Slug functions. To test it, we used a vimentin promoter-luciferase construct (Virtakoivu et al., 2015) . First, we have shown that co-expression of the vimentin promoter-luciferase and Slug significantly increased activity of vimentin promoter, which was further enhanced upon TGFb (positive control) and PRL treatment ( **Fig. 1H** ) suggesting that PRL activates Slug. Next, we have co-expressed the vimentin promoter-luciferase construct and Slug in T47D PAK1 clones treated with either vehicle, TGFb or PRL. We have shown that pTyr-PAK1 significantly increased PRL-dependent Slug activity ( **Fig. 1I )** .

In the present study, we demonstrated that tyrosyl phosphorylated PAK1 stimulates tumor growth in mice. In cancer, EMT is associated with tumor initiation, invasion, and metastasis (reviewed in (Brabletz et al., 2021; Pastushenko & Blanpain, 2019) ). Since we have previously shown that PAK1, in response to PRL, increases the breast cancer cells migration and invasion *in vitro* (Hammer & Diakonova, 2016; Hammer et al., 2015; Hammer et al., 2013; Rider et al., 2013) ; reviewed in (Hammer & Diakonova, 2015) and (Carrasco-Ceballos et al., 2023)) as well as metastasis *in vivo* (Hammer & Diakonova, 2016) , we focused the present study on the role of pTyr-PAK1 in regulation of EMT. We showed here that PRL treatment of T47D cells stably overexpressing PAK1 WT causes decrease of E-cadherin expression and appearance of vimentin in contrast to T47D clones stably overexpressing either GFP or phospho-tyrosyl-deficient PAK1 Y3F mutant. pTyr-PAK1 also promotes cleavage of cell surface E-cadherin (“ecdodomain shedding”), the presence of cleaved E-cadherin fragment in the conditioned media after PRL treatment of PAK1 WT cells was confirmed by mass spectrometry. pTyr-PAK1 increases mRNA levels of Snail, Slug and Twist 2 resulting to decrease expression of E-cadherin. Additionally, pTyr-PAK1 stimulates expression of vimentin via significant enhancement Slug activity. Altogether, our current data provide insight into the mechanism of PRL- and PAK1-stimulated EMT of breast cancer cells.

## Methods

**Cell culture**

Human breast cancer T47D cells stably overexpressing GFP, PAK WT, and PAK1 Y3F were described previously (Hammer et al., 2013) and maintained in RPMI 1640 medium (Corning Cellgro, Corning Inc.) supplemented with 10% fetal bovine serum (Sigma Aldrich). TMX2-28 cells (a variant of the MCF-7 cells (Fasco et al., 2003) ) and their clones stably overexpressing GFP, PAK1 WT or PAK1 Y3F were described previously (Rider et al., 2013) and maintained in DMEM supplemented with 10% FBS. T47D cells were purchased from ATCC and TMX2-28 were kindly donated by Dr. Eisenmann (University of Toledo, OH).

**Reagents**

The cDNA encoding luciferase vimentin promoter were a gift from Dr. Ivaska (Turku Centre for Biotechnology, Turku, Finland) and described in (Virtakoivu et al., 2015) . Myc-tagged Slug in pcDNA was purchased from Addgene (#31698). Monoclonal anti-Snail (C15D3) and anti-Slug (C19G7), monoclonal anti-E-cadherin (32A8) (E-cadherin EC) and monoclonal anti-E-cadherin (2E10) (E-cadherin IC) were from Cell Signaling. PE-conjugated E-cadherin antibody for FACS analysis were from BioLegend (#324105). Monoclonal anti-vimentin V9 were from Invitrogen. Monoclonal anti-γTubulin were from Sigma Aldrich. Polyclonal anti-Twist2 were from GeneTex. Human PRL was purchased from the National Hormone and Peptide Program (Dr. Parlow, National Institute of Diabetes and Digestive and Kidney Disease, Bethesda, MD, USA). TGFβ was from PeproTech and PMA was from Sigma Aldrich.

***In vivo* experiments**

TMX2-28 clones stably overexpressing GFP, PAK WT or PAK1 Y3F were inoculated directly into mammary fat pad of NSG (NOD/SCID/IL2Rgamma) female mice. hPRL (20 µg/100 µl) was injected subcutaneously every other day for 8 weeks and tumor volumes were measured. 10 mice were used for each clone. Mouse experimental procedures were performed in the Animal Research Core of Lerner Research Institute, Cleveland Clinic (Dr. Lindner), and were approved by the Institutional Animal Care and Use Committee, Cleveland Clinic.

**Immunofluorescence and Western blot analysis** were performed as previously described (Hammer & Diakonova, 2016; Hammer et al., 2015; Hammer et al., 2013; Rider et al., 2013) .

**Flow cytometry**

T47D cells expressing GFP, PAK1 WT, or PAK1 Y3F were treated either with 500ng/ml PRL or vehicle for 3 days. Cells were fixed in suspension with 4% formaldehyde and incubated with PE-conjugated E-cadherin antibody (BioLegend, #324105). Fluorescent intensity of surface E-cadherin was analyzed with Becton Dickson FACScalibur fluorescence activated cell sorter and FloJo software and plotted.

**E-cadherin cleavage**

T47D PAK1 WT cells were treated with PRL (500 ng/ml) for 0, 24, 48 and 72 hr. Conditioned media (CM) was concentrated using Amicon Ultra-4 centrifuge filter units. The solubilized proteins were separated by SDS-PAGE followed by immunoblotting with the indicated antibodies. For mass spectrometry analysis, cells were treated as above and the CM was concentrated and subjected to SDS-AGE. The gel was stained with Coomassie blue and the section of gel between 72 kDa and 100 kDa was excised and sent to the Ohio State Mass Spec&Proteomics (MS&P) core for tandem mass spectrometry (MS/MS) analysis (the MS&P project was supported by NIH P30 CA01658 grant).

**Real-time RT-PCR**

Real-time RT-PCR was performed as described previously (Rider et al., 2013) . The primer sequences were as follows: E-cadherin fwd, 5’-CACCTGGAGAGAGGCCGCGT-3’ backward, 5’-TGGGAAATGTGAGCAATTCT-3’; Snail fwd, 5’-AAGATGCACATCCGAAGCCA-3’ backward, 5’-CTCTTGGTGCTTGTGGAGCA-3’; Slug fwd, 5’-CTCAGCTCAGGAGCATACAG-3’ backward, 5’-GACTCACTCGCCCCAAAGATG-3’; Twist1 fwd, 5’-CTGCCCTCGGACAAGCTGAG-3’ backward, 5’-CTAGTGGGACGCGGACATGG-3’; Twist2 fwd, 5’-CGCTACAGCAAGAAGTCGAGC-3’ backward, 5’-GCTCAGCTTGTCAGAGGG-3’ Real-time RT-PCR was performed using IQ SYBR Green Supermix (Bio-Rad). Glyceraldehyde-3-phosphate dehydrogenase was used as the control (house-keeping) gene. All qRT-PCR reactions were performed in triplicate. Each experiment was repeated at least 3 times.

**Vimentin promoter activity**

T47D clones were co-transfected with the vimentin promoter - Luciferase reporter plasmid (VP-Luc), a pCH110 plasmid containing a functional lacZ gene (Virtakoivu et al., 2015) , and either pcDNA vector or pcDNA-Slug-HA (Addgene). The cells were deprived for 24hr, then treated with either vehicle, PRL (500ng/ml), or TGFβ (5ng/ml) for an additional 24hr. The cells were lysed, and luciferase activity was measured using Luciferase assay kit (Promega) according to the manufacturer’s protocol. Luciferase values were corrected for transfection efficiency by determining the ration of luciferase activity to β-galactosidase activity. Each transfection was performed in triplicates. The experiments were repeated at least 3 times.

**Statistical analysis**

Data from at least 3 separate experiments per each condition were pooled and analyzed using 1-way ANOVA plus Tukey’s honest significant difference test. Differences were considered to be statistically significant at P < 0.05. Results are expressed as the mean +/- SE.

## Extended Data

Description: List of identified peptides in deprivation media (control). Resource Type: Dataset. DOI: 10.22002/cszn0-djq35

Description: List of identified peptides after PRL treatment. Resource Type: Dataset. DOI: 10.22002/dta3p-86185

Description: List of identified peptides after the prolactin treatment. Resource Type: Dataset. DOI: 10.22002/gqb75-n2x19

Description: The text file describing the extended data.. Resource Type: Text. DOI: 10.22002/rn47s-4cn39

Description: Summary of identified peptides in all 3 conditions. Resource Type: Image. DOI: 10.22002/6jxff-2g375
